# Research productivity and main publishing institutions in Côte d’Ivoire, 2000–2016

**DOI:** 10.1186/s12992-018-0406-1

**Published:** 2018-08-23

**Authors:** Jasmina Saric, Jürg Utzinger, Bassirou Bonfoh

**Affiliations:** 1 Swiss Tropical and Public Health Institute, P.O. Box, CH-4002 Basel, Switzerland; 2 University of Basel, P.O. Box, CH-4003 Basel, Switzerland; 3 Centre Suisse de Recherches Scientifiques en Côte d’Ivoire, 01 BP 1303, Abidjan, 01 Côte d’Ivoire

**Keywords:** Bibliography, Content analysis, Côte d’Ivoire, Publishing, PubMed, Research productivity, Web of science

## Abstract

**Background:**

The research productivity of countries commonly grouped within sub-Saharan Africa is as diverse as their cultural, economic, linguistic, political, and social profiles. While South Africa has been the science hub on the subcontinent for decades, publishing original research articles in the thousands, Mauritania struggles to have a single publication in international indexed journals in any given year. Detailed country-specific accounts on the co-evolution of research productivity and demographic and economic indicators from sub-Saharan Africa are lacking and render an accurate evaluation and cross-country comparison of internal research progress challenging.

**Methods:**

We assessed the research productivity of Côte d’Ivoire, a francophone West African country that has gone through considerable socio-political unrest, for the period 2000–2016, and determined the main publishing institutions. We considered original research articles extracted from PubMed and Web of Science Core Collection, emphasizing life sciences and biomedical sciences.

**Results:**

We found the quantity of publications doubling from 4.1 to 8.5 per million population and the ‘total product’ – a measure for quantity and quality of published articles – rising from 0.8 to 22.1 per million population between 2000 and 2016. Since 2010 there was a marked increase in the proportion of English publications and a concomitant drop in the proportion of articles with Ivorian first and last authors. The percentage of foreign author contribution increased from 38.7% in 2000 to 71.6% in 2016, suggesting an ‘internationalization’ of the country’s research production and output. Mixed authorship compared with ‘Ivorian only’ showed higher representation in journals with an official impact factor by Web of Science with proportions of 73% versus 28% for 2008 and 91% versus 45% for 2016. Two universities and university hospitals and three autonomous research institutions were consistently among the top 10 institutions publishing peer-reviewed material in three selected years (2000, 2008, and 2016). The main features of the most successful publishing institutions were research staff size, diversification of research portfolio and funding, multiple research bases across the country, and established and productive partnerships with foreign institutions.

**Conclusion:**

Since the turn of the millennium, research productivity in Côte d’Ivoire has steadily grown at an above regional and global rate despite recurring economic pressures and sociopolitical unrest. We have observed benefits of internationalization throughout this current analysis reaching from improved publishing standards to increasing resilience of research institutions in times of crisis.

## Background

National and institutional hotspots of research productivity and scientific excellence on the African continent are important poles for international funders, enhancing visibility, and boosting local and regional education, research, and innovation. Based on research output between 1996 and 2005, 10 African countries (i.e., Egypt, Ethiopia, Kenya, Morocco, Nigeria, Senegal, South Africa, Tunisia, Uganda, and Zimbabwe) were defined as Tier I publishers with the highest research productivity in Africa (> 75 PubMed publications per year) [1]. However, in the meantime, the geopolitical landscape on the continent has changed considerably. While some countries in sub-Saharan Africa have gained prosperity, economic development, and sociopolitical stability, others were left with an even more fragile foundation than before – as aftermath of the Arab spring, for instance. Unfortunately, only few studies prior to 2007 offer insight into the quantity and quality of the national research output within Africa [2–7]. Reports for the most current decade are even scarcer [8, 9]. Francophone, hispanophone, and lusophone nations, in particular, have often been below the radar of the international research community due to a language bias toward English articles on the main international publishing and referencing databases in the life sciences and biomedical sciences.

Although research productivity is being commonly stratified to population size and gross domestic product (GDP), only a small proportion of the published work has attempted to interpret findings in relation to the geopolitical background of a given country. In Libya, for instance, a drop in biomedical publications between the two periods 1986–1996 and 1997–2007 was suggested to reflect the exodus of foreign medical professionals, coupled with a paucity of research culture among Libyan doctors [7]. A correspondence in *The Lancet* in 2003 presented that the share of biomedical publications by the Arab world was less than 1% of the global output despite its relative wealth [10]. The main reasons outlined were the large proportion of military spending in relation to research and development (R&D), health and economic hardship (e.g., Palestine, Somalia, and embargo on Iraq and Libya), and multiple conflicts across the region (e.g., Arab–Israeli, Lebanon, Kuwait, and Iraq). In particular, the war and the post-war period in Kuwait and Lebanon in the early 1990s seemed to strongly influence the publishing behavior of the scientific community, as shown in a decline of publications during the war and a post-war rise [10]. More recent analyses on the determinants of productivity in health research in Africa identified GDP to be a significant predictor, and private health expenditure a marginally significant predictor, of publication quantity, according to binomial analysis [11].

Sub-Saharan Africa is preparing to leap into a more central position in the international research arena. Major international investments are being made to foster large Africa-led programs and pan-African initiatives. The European Union (EU) is boosting research with transregional, continental, or global character via the Pan-African Program since 2014 and also offers separate Horizon 2020 work programs. The European and Developing Countries Clinical Trials Partnership (EDCTP), established in 2001, is funding research for prevention and treatment of poverty-related infectious diseases in sub-Saharan Africa including fellowship programs. The US National Institutes of Health (NIH) have increased investment in Africa with a major allocation of funding directed toward a second round of awards for genomics studies (H3Africa) in 2013, and the World Bank, in 2014, has invested into centers of excellence in seven countries across West and Central Africa.

Major single-country financing schemes include the National Centre of Competence in Research (NCCR) North–South program, facilitated by the Swiss National Science Foundation (SNSF) with additional support from the Swiss Agency for Development and Cooperation (SDC). This program run from 2001 to 2014 and aimed to generate knowledge for sustainable development in sub-Saharan Africa and other low- and middle-income countries [12]. In 2007, the “Programme d’Appui Stratégique à la Recherche Scientifique en Côte d’Ivoire” (PASRES) was initiated, based on the SNSF model to competitively finance research and foster scientific excellence in Côte d’Ivoire. The Wellcome Trust in the United Kingdom has funded the 5-year African Institutions Initiative (AII) in 2009, aiming to build health research capacity in Africa [13], and is currently financing a second phase East–West African collaboration scheme across 11 consortia (DELTAS) [14], together with the UK Department for International Development (DFID), the New Partnership for Africa’s Development (NEPAD), and the African Academy of Sciences (AAS). Moreover, research partnerships between India, China, and Africa, to tackle diseases of poverty, are gaining traction [15, 16].

Knowing the institutional or national research productivity from previous years, and the capacity and efficacy in conducting larger research programs, may offer insights on how well research funds are invested in a given country [13]. It may also encourage the home governments to step up the expenditure for domestic R&D, since a considerable amount of research funding for sub-Saharan Africa still stems from extra-continental funding bodies.

Côte d’Ivoire was classified as low-level Tier IV publishing country initially with 2–10 PubMed publications per year for the period 1996–2005, and rose to become a top ‘Quintile 1’ publisher with more than 1000 articles for the total period 2000–2014 [1, 11]. Côte d’Ivoire has experienced civil war and continuous political turmoil since the late 1990s, accompanied by economic downfall. Indeed, Côte d’Ivoire was one of the most prosperous nations in the West African region until the mid-1990s. Although the second civil war (also referred to as electoral crisis) was officially terminated in 2011 and the Western media has hailed Côte d’Ivoire in recent years as the ‘investors darling of West Africa’, stark national divisions across ethnic groups and the regular flaring up of violence still haunt the country nowadays. However, despite recurring, severe interruptions to the most basic operations within the country, Côte d’Ivoire has managed to maintain strong ties with international research collaborators and transnational consortia to continue R&D activities during this period; in some institutions even during the peak of post-electoral violence in 2010 that lead to half a million displaced people and a 2-year lasting closure of the university system across the entire country [17].

The objective of the current study is to present an assessment of the research productivity and profile of Côte d’Ivoire for the period 2000–2016, with an emphasis on life sciences and biomedical sciences, and with a view to national demography, economic performance, and political background. Moreover, we aimed to identify the most productive publishing institutions during this period as well as resilience factors during times of crisis.

## Results

### Trends in research productivity from 2000 to 2016

**Fig. 1:**
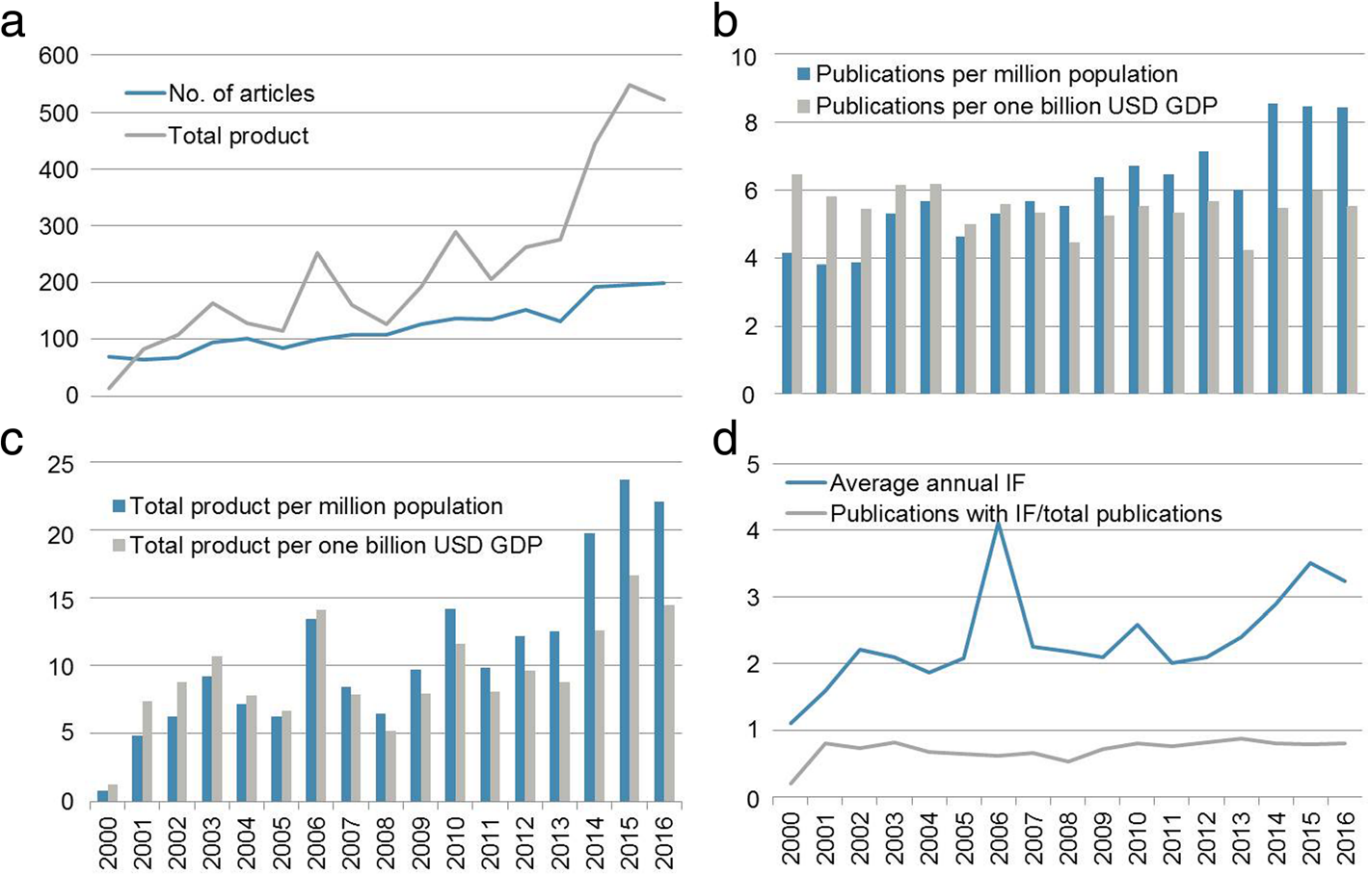
National research productivity in Côte d’Ivoire from 2000 to 2016, as assessed by quantity and quality indicators of original research articles, retrieved from PubMed and Web of Science, by year. **a** Number of original articles and total product (sum of IF*number of articles per journal) by year; **b**, number of original articles normalized to population and GDP; **c** total product normalized to population and GDP; and **d** average annual IF and proportion of publication published in a journal with IF versus total publications. Data on population and GDP were taken from the World Development Indicators put forth by the World Bank (https://data.worldbank.org/data-catalog/world-development-indicators). GDP, gross domestic product; IF, impact factor

The quantity of published original research articles, recovered from PubMed and Web of Science Core Collection, with at least one Ivorian institution affiliation almost tripled between 2000 and 2016; from 69 to 200, with a mean of 121.8 and a median of 108.0 (Fig. 1a). The total product – a measure of quantity and quality, based on the impact factor (IF) and the number of publications for a given journal (n) (**∑ IF**_**j**_**x n**_**j**_) [18] – experienced a marked increase during the 17-year study period from 13.2 in 2000 to 523.0 in 2016 (mean 228.9, median 193.1) (Fig. 1a). When stratified by population, the quantity of publications doubled from 4.1 in 2000 to 8.5 publications per million population in 2016 (mean 6.0, median 5.7). However, normalized to GDP, the publishing quantity showed a slight decline across the time period (6.4 in 2000 and 5.5 in 2016, mean 5.5, median 5.5) (Fig. 1b). For the total product, normalization to population and GDP showed a strong increase from 0.8 to 22.1 per million population (mean 11.0, median 9.7). A similar upward trend was observed for normalization to GDP from 1.2 to 14.5 per 1 billion USD (mean 9.4, median 8.8) from 2000 to 2016 (Fig. 1c).

The average IF as separate measure increased from 1.1 in 2000 to 3.2 in 2016 (mean 2.4, median 2.2) with a peak in 2006, which is also evident in the total product, albeit less pronounced. The 2006 outlier is driven by a single publication in the *New England Journal of Medicine* with an IF of 51.3 in that year. The ratio of articles with IF to total number of articles did not show a clear trend. The lowest value was observed in 2000, followed by some of the highest values from 2001 to 2003 and from 2010 onwards with a drop in-between (Fig. 1d).

**Fig. 2:**
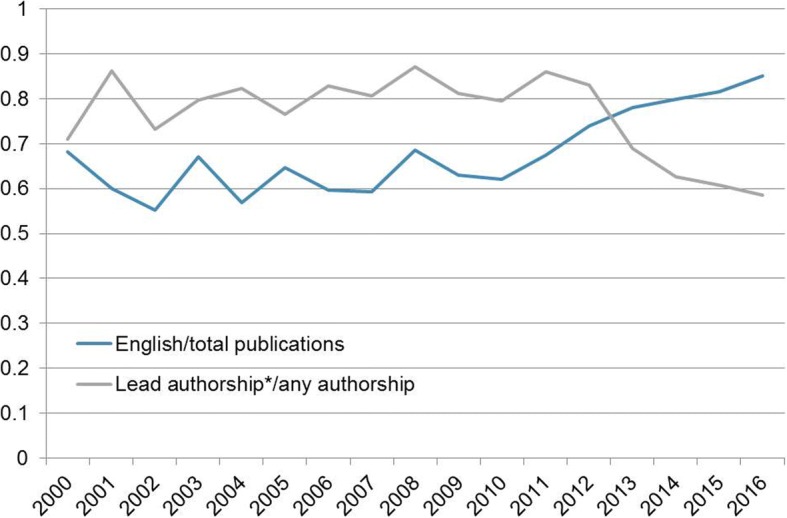
Annual ratio of English versus total publications and annual proportion of articles with Ivorian lead author position (defined as first and/or last or multiple including first and/or last) versus total articles. Original research articles retrieved from PubMed and Web of Science with, at least one Côte d’Ivoire affiliation, for the period 2000–2016, were included in the analysis

Fluctuating during the first decade (2000–2009), the ratio of English to total publications increased steadily from 2010 onwards (from 0.62 in 2010 to 0.85 in 2016), while the ratio of lead author position (first and/or last or multiple including first and/or last) to total author positions experienced the inverse, decreasing in particular between 2011 and 2016 from 0.86 to 0.59 (Fig. 2).

### Dynamics of research domains from 2000 to 2016

**Fig. 3:**
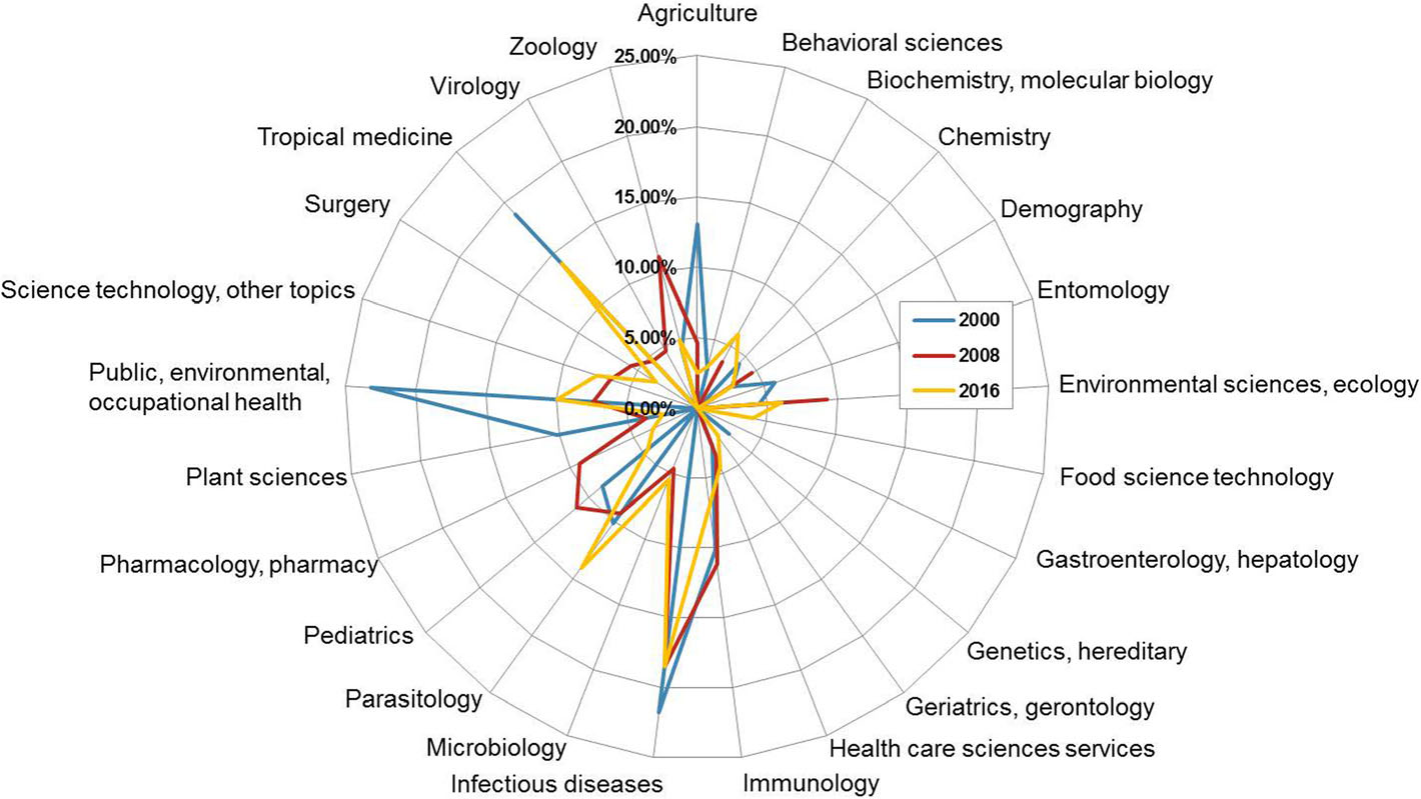
Web of Science analysis of the top 25 represented research areas in Côte d’Ivoire for 2000, 2008, and 2016

Using Web of Science analytical tools for research domains including all original publications from 2000 to 2016, we retrieved the proportion of the top 25 areas published in each year. Over the 17-year study period, a total of 60 different research areas were identified, of which six were present in each year, namely (i) immunology, (ii) infectious diseases, (iii) parasitology, (iv) pediatrics, (v) public, environmental, and occupational health, and (vi) zoology. Of these, the infectious diseases area was the most popular research domain across all years, except in 2006, when pediatrics was the single most important research area. Agriculture; pharmacology and pharmacy; and tropical medicine were represented in 16 and microbiology and plant sciences in 15 out of 17 years analyzed. A direct comparison of the 25 most popular areas of research in 2000, 2008, and 2016 demonstrated a decrease of agriculture; entomology; genetics and heredity; plant sciences; and public, environmental, and occupational health, in favor of food science technology; science technology and other topics; and biochemistry and molecular biology by 2016. In 2008, a temporary increase in environmental sciences and ecology; zoology; pediatrics; and pharmacology and pharmacy, and a temporary decrease in tropical medicine were further observed (Fig. 3). Most areas did not show any obvious time-dependent trend but more or less regular fluctuations. However, there were some science domains that were represented in either earlier and later years only. Entomology, for example, was listed among the 25 main domains per year for each year between 2000 and 2012 but not in any of the subsequent years. Similarly, demography and pathology showed presence mostly before 2012, while dentistry and oral surgery was represented only during 2000 and 2010. The domains of mathematics; urology and nephrology; and veterinary sciences were only found in even earlier years (2000–2002 for mathematics; 2001, 2003, and 2007 for urology and nephrology; and 2001, 2003, and 2005 for veterinary sciences). In contrast, nutrition dietetics was represented four times between 2009 and 2016, while food science technology grew into one of the more popular science domains from 2006 onwards, represented in eight years.

### Main Ivorian publishing institutions and contribution of international partners

**Fig. 4:**
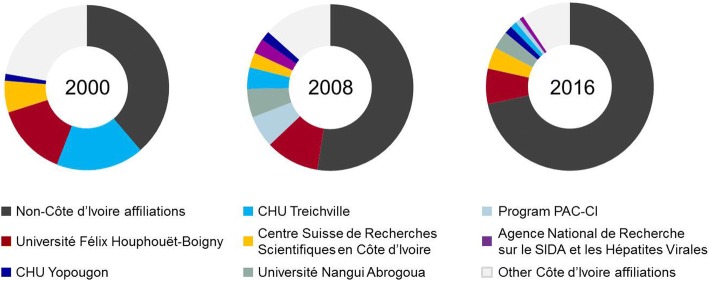
Proportion of contribution of the seven major Ivorian publishing institutions and external affiliations identified during the period 2000–2016. A modified scoring method based on Keiser et al. [19] for the selected years 2000, 2008, and 2016 was applied to rank each Ivorian affiliation. Seven affiliations were found in the top 10 ranks in, at least two of the three assessed years, and were taken forward for full analysis of the entire 17-year period

Institutions in Côte d’Ivoire that were associated with original research articles found on PubMed and/or Web of Science, consisted of (i) the Universities Alassane Ouattara, Félix Houphouët-Boigny, Jean Lorougnon Guédé, Nangui Abrogoua, and Péléforo Gon Coulibaly; (ii) ministries (e.g., Ministry of Public Health and Hygiene, and Ministry of Agriculture); (iii) non-profit actors, including the Centre Suisse de Recherches Scientifiques en Côte d’Ivoire (CSRS), Centers for Disease Control and Prevention (CDC) Abidjan, Institut Pasteur de Côte d’Ivoire, and the World Health Organization (WHO); (iv) private sector such as Novartis, GlaxoSmithKline, Sanofi Pasteur offices in Abidjan; and (v) others (e.g., hospitals, programs/projects and research institutes that are generally listed as separate institutes without a ministry or university as superordinate or co-affiliation). In 2000, a total of 40 different Ivorian affiliations were identified, rising to 42 in 2008 and 63 in 2016. After detailed scoring of all Ivorian affiliations in 2000, 2008, and 2016, seven top publishing institutions were identified that were present among the top 10 in at least two out of three years assessed; namely, the ‘Agence National de la Recherche sur le SIDA et les Hépatites Virales’ (ANRS), Centre Hospitalier Universitaire (CHU) Treichville and CHU Yopougon, CSRS, Program PAC-CI, Université Félix Houphouët-Boigny (formerly Université de Cocody-Abidjan), and Université Nangui Abrogoua (formerly Université d’Abobo-Adjamé). Other trends that were revealed by our in-depth analysis for the three selected years was a steady increase in scores for non-Ivorian affiliations (pooled), a decrease of the proportion of Ivorian affiliations other than the top seven, and a consistently strong presence of Université Félix Houphouët-Boigny under the two top ranks (Fig. 4).

The proportion of the contribution of non-Ivorian affiliations almost doubled during the period from 2000 to 2016; from 38.7% in 2000 to 52.5% in 2008, and 71.6% in 2016, while the proportion of ‘other’ affiliations decreased from 22.3% in 2000 to 13.4% in 2008 and 9.4% in 2016. The proportion of publications with exclusively Ivorian affiliations versus all publications (at least one Ivorian affiliation) lacked a clear trend. For 2000 and again in 2016, the proportion of ‘Ivorian only’ publications was 0.22 (15/69 in 2000 and 44/200 in 2016), for 2008, the proportion was much higher (0.43; 46/108). To assess the impact that the involvement of at least one foreign author may have, we identified the ratio of journals where the research was published, distinguishing between journals that have an official IF according to Web of Science in a given year, versus total journal published in for each category of ‘Ivorian only’ and mixed nationality publications. For 2000, one third of all ‘Ivorian only’ publications were published in journals that possess an IF, while only half as much (15%) of publications by mixed authorships were published in journals with an IF. The same comparison for 2008 is 28% for ‘Ivorian only’ versus 73%, and for 2016 45% versus 91%.

**Fig. 5:**
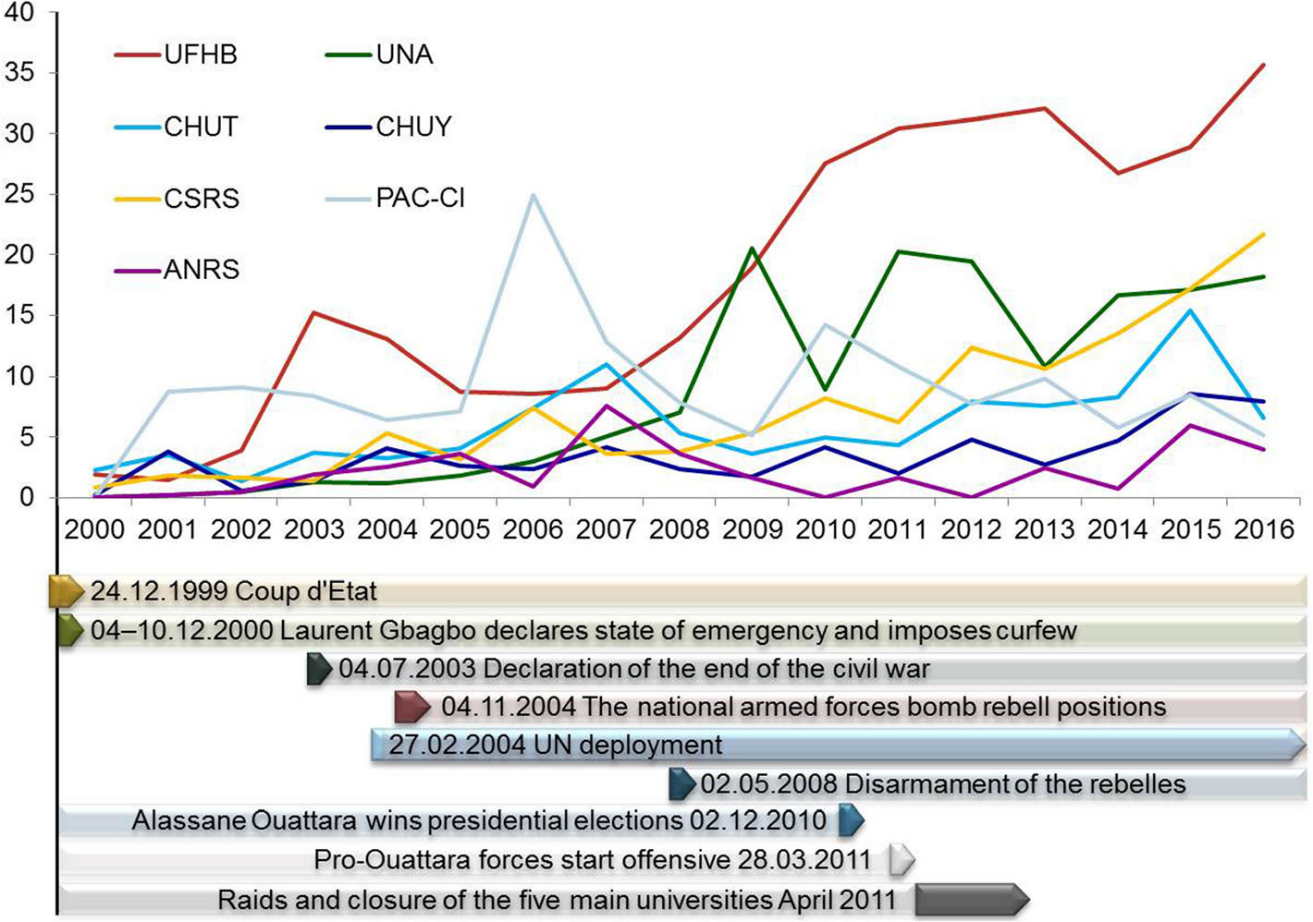
Co-evolution of the productivity of the seven top publishing institutions in Côte d’Ivoire (top) in relation to the main geopolitical events between 2000 and 2016 (bottom). ANRS, Agence Nationale de Recherche sur le SIDA et les Hépatites Virales; CHUT, Centre Hospitalier Universitaire Treichville; CHUY, Centre Hospitalier Universitaire Yopougon; CSRS, Centre Suisse de Recherches Scientifiques en Côte d’Ivoire; PAC-CI,  Program PAC-CI; UFHB, Université Félix Houphouët-Boigny; UNA, Université Nangui Abrogoua

The leading Ivorian publishing institution in the three years studied in greater detail – i.e., Université Félix Houphouët-Boigny – was represented with 14.2% in 2000, 10.4% in 2008, and 6.8% in 2016. This university was also the most prolific publishing institute among the selected top seven, when looking at the whole period being ranked top in 10 out of the 17 years assessed (Fig. 5). The second university present among the top scorers – i.e., Université Nangui Abrogoua – was found among the top two positions in five years, being on top once in 2009. The Program PAC-CI was present among the top two scores nine times of which eight times were before 2008. CSRS scores increased steadily throughout the 17-year analysis period with consistent representation among the top three ranks from 2012 onwards finishing second in 2016.

Averaging the scores for all 17 years, the following order of ranks was found: 1st, Université Félix Houphouët-Boigny; 2nd, Program PAC-CI; 3rd, Université Nangui Abrogoua; 4th, CSRS; 5th, CHU Treichville; 6th; CHU Yopougon; and 7th ANRS. Of note, the Program PAC-CI at the ANRS research site is physically located at the CHU Treichville. Although all three affiliations are often co-associated in the publications, they are also represented separately and/or together with a different set of affiliations. All three were therefore treated as separate publishing institutions and scored separately.

## Discussion

### Global context

Official figures for total publications per million inhabitants for 2008 and 2014 from the UNESCO Science Report for 2030 [20], revealed a global growth by 15%; 32% for lower middle-income countries, and 33% for sub-Saharan Africa, including South Africa. In comparison, Côte d’Ivoire, for the same period, showed a striking 55% of growth based on our own data. The previous UNESCO Science Report 2010 [21] did not report on the publications per population size, presenting growth based on total publications between 2002 and 2008, and an income-based classification of countries had not been introduced by the time the report was established. However, based on population data from the World Bank, population stratified growth between 2002 and 2014 can be extrapolated as 51% globally and as 107% for sub-Saharan Africa, including South Africa. The data we extracted for Côte d’Ivoire for the same period suggest again above-global and regional average growth by 122%, despite multiple periods of turmoil. However, it has to be pointed out that the methodology from the UNESCO report may vary from ours in terms of databases used and documents included. Indeed, looking at the scientific publications by country annex of the UNESCO report, the average numbers for Côte d’Ivoire seem to be slightly higher than ours. Growth of the UNESCO assessment of Côte d’Ivoire was therefore ‘only’ 44% between 2002 and 2014, which is based on a notably higher number of publications retrieved by UNESCO in 2002 compared with our analysis (111 versus 67).

Comparing research productivity in Côte d’Ivoire with other African countries is equally challenging owing to the scarcity of studies available and the inconsistency of study designs with respect to performance indicators, databases searched, article types included, time periods surveyed, and scientific domains assessed [2–9]. Using article quantity normalized to GDP and population offers, thus far, the best comparative base as information on %GDP spent on R&D and institutional funding details are difficult to obtain, as experienced in the current analysis and work pursued before. An assessment of the Maghreb zone, including Libya, Morocco, and Tunisia, for instance, retrieving all biomedical publications from PubMed during the period 2001–2006 placed Tunisia at the top end of performance with 20.4 publications per million population and 7.2 publications per 1 billion USD GDP per year. For Libya, approximately 10 times less (2.4) publications per million population and 20 times less (0.4) publications per 1 billion USD GDP per year were reported [4]. The Tier assessment of biomedical PubMed publications across Africa conducted for 1996 to 2005 found South Africa and The Gambia at the top end for publications normalized to population (median between 20 and 30), while Cameroon, Togo, Malawi, Nigeria, and Guinea ranked at the bottom end (median between 0 and 5) per million population per year for the period 1996–2005. Normalized to total GDP, The Gambia was positioned again at the top (median between 60 and 80 publications per 1 billion USD per year), while South Africa, Mali, and Benin were found among the poorest performing (median between 0 and 5) [1]. Averaged Côte d’Ivoire figures for 2001–2005 are 4.7 publications per million population and 5.7 publications per 1 billion USD per year, which positions the country somewhere in between the best and the worst performing nations across the Maghreb zone and sub-Saharan Africa. However, in contrast to those two multi-country assessments based on research articles extracted from PubMed, our design for an in-depth analysis of research productivity of Côte d’Ivoire included articles from the Web of Science Core Collection, in addition to PubMed, which accounted for 1/6th to 1/3rd of the total publications retrieved per year. Hence, the overall quantity of publications in the current study is expected to be relatively higher.

### Evolution of publishing activities in Côte d’Ivoire

Of note, a direct comparison was possible along the timeline of research productivity for Côte d’Ivoire. Here, somewhat of a contradiction was observed when looking at the stratification to population size versus stratification to GDP. Both the publication quantity and the total product increased from 2000 to 2016 for the same population size. While the total product also increased when stratifying to GDP, the publication quantity did not show such a timeline-dependent increase per GDP. Since the total product is a measure for both quantity and quality, one possible conclusion is that resources invested in R&D have impacted on quality rather than quantity and that the increase of the total product along the time-period assessed is driven by the quality indicator (IF) mainly. Unfortunately, there are no detailed %GDP data for R&D expenditure openly available for Côte d’Ivoire, which challenges a more comprehensive interpretation of this phenomenon. However, the higher weight of the quality component is further underlined by the unchanged ratio of publications with IF versus total publications that accompanies the increase of the average annual IF and the proportion of English publications along the timeline assessed. It is not the quantity of journals with IF chosen that seems to have changed, but the average IF of those journals seems to have increased, which may suggest that good publishing practices (that include publishing in indexed journals) are implemented by an ever more quality-conscious and internationally connected scientific community but may not be reaching the bulk of researchers in Côte d’Ivoire yet.

The relative increase in English publications also coincided with a drop in the proportion of articles published by Ivorian researchers in lead author positions (first and/or last author) that became evident from 2011 onwards (Fig. 2). Considering the increasing scores of non-Ivorian affiliations along the timeline assessed on top of that (Fig. 4), we might be witnessing a certain loss of ownership of the research by Ivorian research institutions or simply a phase of adaptation and transition to a new publishing culture using the growing international and transnational networks and consortia available. Increasing international collaborations, involvement in larger, better funded, and hence, often more impactful research, multinational consortia, and other forms of enhanced international visibility are welcomed developments to the transition that seems to be going on in Côte d’Ivoire [22–26]. In addition, the increasing internationalization seems to directly impact on the quality of the journals chosen for publishing the research. Less than one third of all ‘Ivorian only’ publications were published in journals that possessed an IF in 2008, while 73% of mixed publications did so. For 2016 the respective percentages were 45% and 91%.

The research portfolio of Côte d’Ivoire showed a high consistency among the most popular scientific areas along the decades assessed. The classical tropics and subtropics disciplines infectious diseases, parasitology, tropical medicine, and to some extend immunology and pediatrics, were strongly represented in most years assessed. Changes in scientific foci observed, seem to have less to do with the sociopolitical environment and more with the global trends in science and R&D funding. The single-disciplinary mathematics, entomology, demography, dentistry, and urology gave way to more laboratory-based and multidisciplinary domains of research such as food science technology, science technology, and biochemistry and molecular biology by 2016 (Fig. 3). The strong downward trend in agricultural sciences between 2000 and 2016 is surprising, considering that the economy of Côte d’Ivoire has been, and continues to be, largely driven by agricultural development. However, since the rating is based on proportions rather than absolute numbers, there are a variety of explaining factors including the shift in science trends, an increasing multidisciplinary environment, and the fact that most agricultural research institutes in Côte d’Ivoire (e.g., Institute de Recherche pour le Development, West Africa Rice Development Association, and CIRAD) were closed during one or more periods of violence between 2000 and 2016 [27].

### Resilience of the main Ivorian publishing institutions

Two universities, two university hospitals, and three research facilities (i.e., one center, one site, and one program) were identified as top publishing institutions during the period 2000–2016. Université Félix Houphouët-Boigny was found to be the top publisher in the majority of the 17 years assessed. Based in Abidjan, the economic capital of Côte d’Ivoire, Université Félix Houphouët-Boigny represents by far the largest university in the country with 13 faculties and approximately 2000 academic staff and 60,000 students. The sheer size and research capacity of this university partly explains the consistent top scores. The second university found among the top publishers, Université Nangui Abrogoua, is also Abidjan-based and consists of four faculties, enrolling approximately 8400 students. Together with the three other main universities across the country (Université Alassane Ouattara in central Bouaké, Université Jean Lorougnon Guédé in central-west Daloa, and Université Péléforo Gon Coulibaly in northern Korhogo), they all experienced an almost 2-year closure due to spill-overs from the post-electoral violence from April 2011 to September 2012, leaving laboratories looted, infrastructure dysfunctional, and researchers and students without a base [27]. While for Université Nangui Abrogoua a major drop in original publications was seen in 2013 (Fig. 4) recovering in subsequent years, the productivity of Université Félix Houphouët-Boigny dropped in 2014 and 2015 and recovered in 2016. The lag in effect suggests that the externally imposed restrictions to actively pursue research in the laboratory or the field coupled with reduced teaching and mentoring duties, might have been compensated with desk-based work. In this scenario, the writing up of publications offers a means to be productive as researcher given that any efforts to obtain funding for a project based or partially based in a fragile context will face additional challenges. The fact that Université Félix Houphouët-Boigny experienced a later drop compared with Université Nangui Abrogoua may be due to its larger research population generating a larger volume of data available per scientist during the closure.

The two public teaching hospitals (CHU Treichville and CHU Yopougon) were established in their current form in the mid-1970s and are associated with the medical faculty of the Université Félix Houphouët-Boigny. However, they are represented as autonomous institutions in most of the published work. CHU Treichville is often co-associated with the prolific HIV/AIDS Program PAC-CI and ANRS, which may have contributed to the high scoring. A similar trend applies to CHU Yopougon, though to a lesser extent. Particularly the pediatric services of CHU Yopougon are frequently associated with the work of the Program PAC-CI and ANRS. To our knowledge, unlike the universities, the teaching hospitals did not experience any significant periods of interrupted services during the post-electoral crisis.

The ANRS office in Abidjan was established in the early-1990s at the CHU Treichville by the French government to promote research on HIV/AIDS and hepatitis. The Program PAC-CI was launched in 1995, readily embedded in ANRS and consists of a team of approximately 100 staff. Despite the relatively small size of the local team, the Program PAC-CI has established itself as one of the top players in publishing original research articles across the country during the 17-year period assessed. Publications in very high IF journals, including the *New England Journal of Medicine* (IF in 2016: 72.4), and consistent presence in intermediate IF journals in the domain of HIV/AIDS (i.e., *AIDS* and the *Journal of Acquired Immune Deficiency Syndromes*), strong ties with University Victor Segalen and INSERM in Bordeaux, France, coupled with a peak of investment in global HIV/AIDS research from 2000 up to 2010, are likely contributors to this success story. Intense lobbying by HIV/AIDS activists headed by Peter Piot, UNAIDS Director at the time, resulted in the inclusion of HIV/AIDS as one of the Millennium Development Goals, leading indeed to the access to a considerable, new funding stream [28]. However, the Program PAC-CI publishing activities started declining after 2011, which may be again an indication of the enforced low-level of research activities that has taken place across the country as a consequence of the widespread post-electoral violence. An equally plausible reason for the drop in research productivity of the Program PAC-CI is the decrease of international funding for HIV/AIDS programs as consequence of the global financial crisis.

A very different scenario emerges for CSRS, which is a public–private research institution of similar size as the Program PAC-CI with approximately 170 researchers and 100 support staff. In contrast to the Program PAC-CI and its associated institutions that published less in 2016 than in 2015, the CSRS continuously increased its publication output along the whole period assessed, finishing as second-most prolific publishing institute in Côte d’Ivoire behind Université Félix Houphouët-Boigny in 2016. In our view, the infrastructure, a well-articulated strategic plan, established governance structure with administrative and scientific boards that meet once or twice a year, and strong international partnerships are the main explanatory factors for the resilience of CSRS [29]. Indeed, resilience toward changing trends in funding is achieved by the diversification of its research portfolio based on eight main domains of research, including parasitic diseases, bio-conservation, nutrition, agriculture, and environmental sciences. A cross-cutting and transdisciplinary approach is generally implemented to work on a given scientific question that includes a strong social science component allowing for a wider understanding of the research setting. Resilience to geopolitical threats, on the other hand, is achieved by the low-key/low-visibility location of the main research station outside central Abidjan and multiple satellite laboratories across the country that offer attractive research bases and that have helped maintaining a minimum of active field- and laboratory-based research activity during the entire post-electoral crisis [17, 30].

At least for CSRS, we know for certain that a strong backing by some of its long-term international partner institutions, along with funding from the Swiss State Secretariat for Education, Research and Innovation (SERI), have contributed to keeping up the research productivity during periods that were restrictive to conducting laboratory and field work [17, 30]. Moreover, the CSRS has benefited from competitively acquired international large-scale and smaller local Swiss funding schemes for education and research (i.e., NCCR North–South, AII, DELTAS, and PASRES). However, those two final points made might also hold true for the other main publishing institutions identified in this work.

The methodology of the current study aimed to capture national research productivity as measured by international indicators and standards. It should be emphasized that on local and regional level, the perception of research productivity may differ quite considerably owing to the research and academic structures prevailing among francophone and lusophone African countries. The Conseil Africain et Malgache pour l’Enseignement Supérieur (CAMES; African and Malagasy Council for Higher Education) that evaluate higher education and research systems dictates a different publishing policy compared with international standards. The most important deviations are the lack of significance accounted to the IF, focusing almost solely on the quantity of publications. The key authorship positions are also defined somewhat differently by CAMES; the first three author positions are the most important ones, as opposed to the first and last author as for international publishing. Both differences pose a conflict to institutions and individual scientists who want to gain international recognition.

## Conclusions

Côte d’Ivoire's scientific output has grown steadily since 2000 at a rate that outperforms regional and global average growth. This growth has been accompanied by high quality standards in publishing that are partially driven by intensified collaboration with international partners. Sociopolitical factors seem to have left the research productivity and quality largely unaffected, with the exception of the 2-year university closure that may have caused a temporary drop for the two largest universities based in Abidjan. Given that only two out of five main universities across Côte d’Ivoire are among the top publishing institutions in the 17-year period assessed in this work, and considering the number of scientists who contribute to the annual research output (~ 2000 at Université Félix Houphouët-Boigny versus ~ 170 at CSRS and less than 100 at Program PAC-CI), the smaller public–private research institutions clearly outperform the purely academic establishments in this country. Well established strategic plans, strong international research partnerships, size, diversity of research portfolio, and potential funders and a solid base with different research hubs across the country, were found to be the main features of the strongest publishing institutions equipping them with means of resilience, and allowing for flexibility in research strategy.

## Methods

To scope out the field of peer-reviewed work on national research productivity on the African continent, the following PubMed search was conducted: [(africa) AND (publishing[Title] OR research productivity[Title] publication*[Title] OR quantity[Title] OR quality[Title] OR bibliometric OR literature) AND pubmed].

### Data sources

A full search was conducted using PubMed and Web of Science Core Collection (Clarivate Analytics) for each year separately from 1st January 2000 to 31st December 2016. PubMed is a platform for life sciences and biomedical sciences specifically, while the Web of Science Core Collection covers for science and technology, social sciences, and arts and humanities, allowing us to broaden the national research profiling. The same search was applied to both databases using [Côte d’Ivoire OR Ivory Coast]. A search across all fields was preferred to an affiliation and/or publication-specific search in order to maximize the yield of relevant data (information on references older than 2011 can be incomplete in PubMed). For the same reasons, the default topic search was preferred to an address-specific search in Web of Science. The initial output of both databases was copied into Excel and screened, discarding duplications between years and between databases.

Of the remaining publications, author position (first, last, middle, or multiple [multiple positions including first and/or last]) language (English/non-English), journal name, publishing details, article type, title of publication, and affiliations were extracted from the original publications. Where original publications were neither openly accessible nor could they be ordered, the corresponding journal homepage or PubMed and Web of Science databases were consulted to extract data. If the relevant data were not available by any of those means, the reference was excluded from further analysis (no more than 10 in total). Only articles with at least one Ivorian institution affiliation and original/full research articles including meta-analyses were further analyzed, excluding case reports, comments, communications, letters, notes, reviews, short reports, and structure reports.

### Performance measures

Performance measures were chosen according to previously published studies on research productivity [4, 18, 31]. The IF of the respective year assessed was extracted from Web of Science and the quantity of articles, the average IF, and the total product (**∑** ***IF***_***j***_***x n***_***j***_), *IF*_*j*_ and *n*_*j*_ being the IF and the number of publications for a given journal [18], were subsequently determined for each of the years 2000 to 2016. This time period was chosen to comprehend the main political and economic changes since 2002, including the onset of the civil war in 2002 and the post-electoral crisis in 2011, and the establishment of a new economic order by the new regime since. Years 2000 and 2001 were assessed to have a pre-crisis baseline. To allow for direct comparison between the 17 years assessed, article quantity and total product were stratified to population size per million population, and GDP per 1 billion USD based on the corresponding World Development Indicators by the World Bank (https://data.worldbank.org/data-catalog/world-development-indicators). The ratios of (i) English versus total publications; (ii) publications with lead author affiliation versus total publications; and (iii) articles published in journals with IF versus total publications were also determined.

### Scoring

To identify the most prolific Ivorian publishing institutions, an overall score of 1 was allocated to each publication, divided by the number of authors and sub-divided by the number of affiliations as previously done by Keiser and colleagues [19]. However, while the study of Keiser et al. was solely concerned about the quantity of representation on a given publication, assessing the geographic distribution of affiliations, the current study aimed at assessing both quantity and quality of the representation across the original articles published. The initial score was therefore multiplied by the IF of the corresponding journal and the respective year for each article being scored, to account for the quality of a representation and the same time to avoid skewing the score toward representations in single-institutional articles and in articles that are in non-indexed journals and/or of low impact.

In order to identify the top publishing institutions between 2000 and 2016 and characterize their evolution along the period assessed, for the years 2000, 2008, and 2016 each Ivorian affiliation was scored and documented. Ivorian publishing institutions that were among the top 10 publishers in at least two of those three years assessed were subsequently scored for the whole period from 2000 to 2016.

Where certain affiliations were sometimes presented with and sometimes without a second, different affiliation, the behavior of those specific pairs was assessed across the time period. If the one of the affiliations appeared on its own in the majority of cases, affiliations were split consistently, otherwise summarized into one single umbrella affiliation.
